# Associations of pathological diagnosis and genetic abnormalities in meningiomas with the embryological origins of the meninges

**DOI:** 10.1038/s41598-021-86298-9

**Published:** 2021-03-26

**Authors:** Atsushi Okano, Satoru Miyawaki, Hiroki Hongo, Shogo Dofuku, Yu Teranishi, Jun Mitsui, Michihiro Tanaka, Masahiro Shin, Hirofumi Nakatomi, Nobuhito Saito

**Affiliations:** 1 Department of Neurosurgery, Faculty of Medicine, The University of Tokyo, 7-3-1 Hongo, Bunkyo-ku, Tokyo, Japan; 2 Department of Molecular Neurology, Graduate School of Medicine, The University of Tokyo, 7-3-1 Hongo, Bunkyo-ku, Tokyo, Japan; 3 Departments of Neuroendovascular Surgery, Kameda Medical Center, 929 Higashi-cho, Kamogawa, Chiba Japan

**Keywords:** Molecular biology, Neuroscience, Diseases, Neurology, Oncology

## Abstract

Certain driver mutations and pathological diagnoses are associated with the anatomical site of meningioma, based on which the meninges have different embryological origins. We hypothesized that mutations and pathological diagnoses of meningiomas are associated with different embryological origins. We comprehensively evaluated associations among tumor location, pathological diagnosis (histological type), and genetic alterations including *AKT1, KLF4, SMO, POLR2A*, and *NF2* mutations and 22q deletion in 269 meningioma cases. Based on the embryological origin of meninges, the tumor locations were as follows: neural crest, paraxial mesodermal, and dorsal mesodermal origins. Tumors originating from the dura of certain embryologic origin displayed a significantly different pathological diagnoses and genetic abnormality ratio. For instance, driver genetic mutations with *AKT1, KLF4, SMO*, and *POLR2A*, were significantly associated with the paraxial mesodermal origin (*p* = 1.7 × 10^−10^). However, meningiomas with *NF2*-associated mutations were significantly associated with neural crest origin (*p* = 3.9 × 10^–12^). On analysis of recurrence, no difference was observed in embryological origin. However, *POLR2A* mutation was a risk factor for the tumor recurrence (*p* = 1.7 × 10^−2^, Hazard Ratio 4.08, 95% Confidence Interval 1.28–13.0). Assessment of the embryological origin of the meninges may provide novel insights into the pathomechanism of meningiomas.

## Introduction

Meningiomas are the most common primary intracranial tumors accounting for 20% of all such tumors. Approximately 69% of meningiomas are benign (WHO grade I), while 29% are atypical (WHO grade II), and 2% are malignant (WHO grade III)^1^. Previous studies have suggested an association between meningioma location and histological grading, with non-skull-base meningiomas displaying more aggressive biological behavior^2–6^.

Molecular genetic investigations have revealed *NF2* gene mutations in approximately 40–60% of sporadic meningiomas^6,7^. Recent studies have reported *TRAF7*, *KLF4*, *AKT1*, *SMO*, *PIK3CA*, and *POLR2A* mutations, all mutually exclusive of *NF2* mutations^8–13^. In these reports, *NF2* mutations and/or loss of chromosome 22 are predominant in meningiomas originating from the cerebral convexity and cerebellar dura and in the spinal canal^8,12,14,15^. On comparing different skull-base locations, most non-*NF2* meningiomas were located on the medial skull base, whereas those on the lateral and posterior skull base harbored *NF2* mutations or loss of chromosome 22^7,8,12,16–19^. Thus, the gene mutations may be potentially associated with anatomical sites.

The meninges might have different embryological origins depending on the anatomical site. Numerous studies on meningeal development in humans strongly indicate three sources of embryogenesis: the neural crest, the paraxial mesoderm, and the dorsal mesoderm^20–25^. These differences in the embryological origin of the meninges are associated with the pathophysiology of various diseases^26,27^.

We hypothesized that driver mutations and meningioma-related pathologies are associated with the meningeal origin. We modified the concept of tumor location in accordance with the embryological origin of different meningeal parts to verify this hypothesis. Thereafter, we comprehensively evaluated the associations between tumor location (embryological origin), pathological diagnosis (histological type), and driver mutations including *AKT1*, *KLF4*, *SMO*, *POLR2A*, and *NF2* mutations. Finally, we evaluated the factors affecting tumor recurrence, such as clinical parameters, embryological origins, pathological diagnosis and genetic mutations.

## Results

### Patient characteristics

A total of 499 meningioma patients who had undergone surgery at the University of Tokyo Hospital, Tokyo, Japan, between January 2000 and June 2017 were included herein. Among them, 269 patients adhered to the study inclusion criteria. This study included 192 women (71%) and 77 men (29%). The patient mean age was 58.0 years (range; 0.1–81.2 years) and mean follow-up duration was 50.4 months (range; 1–199) (Table 1).

**Table 1:** Patient characteristics with clinical, genetic and histological features.

	General Cohort (n = 269)	*SMO* (n = 1; 0.3%)	*AKT1* (n = 28; 10.4%)	*KLF4* (n = 16; 5.9%)	*POLR2A* (n = 17; 6.3%)	*NF2* + *22q loss* (n = 87; 32.3%)	*NF2* (n = 19; 7.1%)	*22q loss* (n = 53; 19.7%)	Not detected (n = 48; 17.8%)
**Age (year) (mean ± SD)**	58.0 ± 2.2	59.9	57.3 ± 2.4	56.2 ± 3.3	51.6 ± 3.2	59.5 ± 1.4	53.7 ± 3.0	61.2 ± 1.8	58.7 ± 0.9
**Sex**									
Female	192 (71.3%)	0 (0)	20 (7.4%)	10 (3.7%)	17 (6.3%)	58 (21.6%)	16 (5.9%)	37 (13.8%)	34 (12.6%)
Male	77 (28.6%)	1 (0.3%)	9 (3.3%)	6 (2.2%)	0 (0)	29 (10.8%)	3 (1.1%)	16 (5.9%)	14 (5.2%)
**Localization**									
Anterior	18 (6.7%)	1 (0.3%)	8 (3.0%)	0 (0.3%)	1 (0.0%)	2 (0.7%)	0 (0)	0 (0)	6 (2.2%)
Central	76 (28.3%)	0 (0)	12 (4.5%)	11 (4.1%)	10 (3.7%)	17 (6.3%)	3 (1.1%)	8 (3.0%)	15 (5.6%)
Lateral	25 (9.3%)	0 (0)	6 (2.2%)	4 (1.5%)	0 (0)	5 (1.9%)	0 (0)	4 (1.5%)	6 (2.2%)
Posterior	28 (10.4%)	0 (0)	2 (0.7%)	0 (0)	2 (0.7%)	11 (4.1%)	2 (0.7%)	6 (2.2%)	5 (1.9%)
ST-med	56 (20.8%)	0 (0)	0 (0)	1 (0.3%)	0 (0)	29 (10.8%)	5 (1.9%)	15 (5.6%)	6 (2.2%)
ST-ant/lat	39 (14.5%)	0 (0)	0 (0)	0 (0)	0 (0)	14 (5.2%)	5 (1.9%)	16 (5.9%)	4 (1.5%)
ST-post/lat	3 (1.1%)	0 (0)	0 (0)	0 (0)	0 (0)	0 (0)	0 (0)	1 (0.3%)	2 (0.7%)
Cerebellar tentorium	24 (8.9%)	0 (0)	0 (0)	0 (0)	4 (1.5%)	9 (3.3%)	4 (1.5%)	3 (1.1%)	4 (1.5%)
**Localization (embryological)**									
Neural crest	144 (53.5%)	0 (0)	6 (2.2%)	5 (1.9%)	4 (1.5%)	57 (21.2%)	14 (5.2%)	38 (14.1%)	20 (7.4%)
Paraxial mesoderm	97 (36.1%)	1 (0.3%)	20 (7.4%)	11 (4.1%)	11 (4.1%)	19 (7.1%)	3 (1.1%)	9 (3.3%)	23 (8.6%)
Dorsal mesoderm	28 (10.4%)	0 (0)	2 (0.7%)	0 (0)	2 (0.7%)	11 (4.1%)	2 (0.7%)	6 (2.2%)	5 (1.9%)
**WHO grade**									
I	243 (90.3%)	1 (0.3%)	27 (10.4%)	16 (5.9%)	17 (6.3%)	76 (28.3%)	18 (6.7%)	43 (16.0%)	45 (16.7%)
II	26 (9.7%)	0 (0)	1 (0.3%)	0 (0)	0 (0)	11 (4.1%)	1 (0.3%)	10 (3.7%)	3 (1.1%)
III	0	0	0	0	0	0	0	0	0
**MIB-1 LI (mean ± SD)**	3.1 ± 0.2	3	2.1 ± 0.7	1.7 ± 0.9	1.2 ± 0.9	3.9 ± 0.4	2.6 ± 0.8	3.6 ± 0.5	2.8 ± 0.5
**Simpson grade**									
1	50	0	5	1	0	15	6	15	8
2	115	0	15	5	9	35	9	18	24
3	30	1	1	4	2	9	3	7	3
4	53	0	7	6	6	16	1	7	11
5	3	0	0	0	0	2	0	1	0
**Follow up (years)**	4.2 ± 0.2	1.3	3.3 ± 0.7	4.3 ± 1.0	3.8 ± 1.0	3.8 ± 0.4	4.0 ± 0.9	4.3 ± 0.5	5.1 ± 0.6
**Recurrence**	51 (19.0%)	1(100%)	3(10.3%)	2 (12.5%)	5 (29.4%)	22(25.6%)	2 (10.5%)	10(19.2%)	6 (12.5%)

*SD* indicates standard deviatation, *ST-med* supra-tentorial-medial, *ST-ant/lat* supra-tentorial-antero-lateral, *ST-post/lat* supra-tentorial-postero-lateral, *LI* labelling index.

### Pathological diagnosis and embryological origins of the meninges

Table1 provides raw data regarding tumor locations, genetic status and clinico-histopathological features.

**Figure 1:**
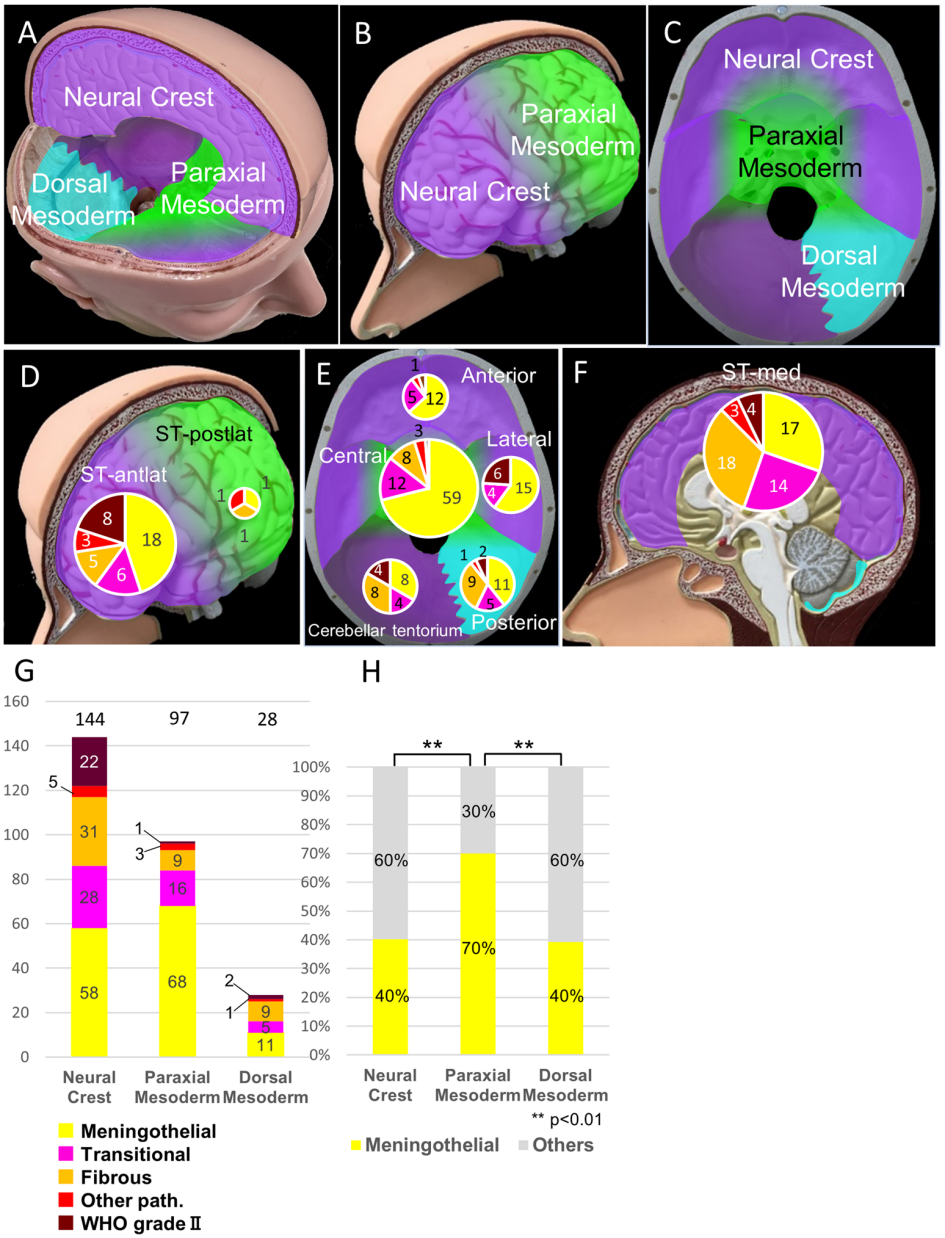
Pathological diagnosis according to embryologically classified anatomical location of meningiomas. (**A**–**C**) Embryological origins of the intracranial meninges. (**D**) Pathological distribution at ST-ant/lat and ST-post/lat. Each pie chart provides a breakdown of the number of each pathological diagnosis. The size of the pie charts reflects the total number of cases of this type of lesion. (**E**) Pathological distribution at the skull-base. The numbers indicate the number of cases for each location. The sizes of the pie chart indicate the total number of cases for each location. (**F**) Pathological distribution at ST-med. The number indicates the number of cases for each location. The sizes of the pie chart indicate the total number of cases for each location. (**G**) The number of pathological diagnoses for each embryologically classified tumor. The bar graph shows the number of pathological diagnoses for each embryologically classified tumor. (**H**) Comparison of the ratio of meningothelial meningioma among embryologically classified tumors. The ratio of meningothelial meningioma is significantly higher in tumors derived from the paraxial mesoderm rather than the neural crest or dorsal mesoderm. (*p* < 0.01, statistically significant on the chi-square test with Bonferroni correction). ST-ant/lat, supra-tentorial-antero-lateral; ST-post/lat, supra-tentorial-postero-lateral; ST-med, supra-tentorial-medial. Figure generated using Microsoft PowerPoint 2016, https://www.microsoft.com.

Figure 1A–C shows a schematic representation of the spatial distribution of the embryological origins of the meninges based on previous reports. Dura with neural crest origin is purple, that originating from the paraxial mesoderm is green, and that originating from the dorsal mesoderm origin is blue. Herein, 144 cases were included in the neural crest group, 97 cases in the paraxial mesoderm group, and 28 cases in the dorsal mesoderm group (Table 1).

Pathological diagnosis revealed WHO grade I meningioma in 243 tumors (90.3%), including 135 of meningothelial, 49 of transitional, and 49 of fibrous types. WHO grade II tumors were detected in 26 cases (9.7%), while no cases presented WHO grade III tumors. The proportion of fibrous meningiomas was relatively higher in the ST-med and posterior skull base. WHO grade II meningiomas were more frequent at ST-ant/lat, ST-med, lateral, and posterior, and meningothelial meningiomas were more frequent at the skull base, especially in the “Central” and “Anterior” regions (Fig. 1D–F). One of the reasons for the small number of WHO grade II cases herein may be that our cohort has a high proportion of skull base meningiomas. In fact, 171 of 269 (63.6%) tumors were located at the skull base.

Regarding the association between pathological diagnosis and embryological origins of the meninges, the proportion of meningothelial meningiomas was significantly higher in patients with lesions originating from the paraxial mesoderm rather than from the neural crest (*p* = 5.5 × 10^–6^) and the dorsal mesoderm (*p* = 2.9 × 10^–3^) (Fig. 1G,H). However, the proportion of fibrous meningiomas was significantly higher among patients with lesions originating from the neural crest rather than the paraxial mesoderm (*p* = 0.01) (Supplemental Fig. 1A). The proportion of WHO grade II meningiomas was higher among patients with lesions originating from the neural crest rather than from the paraxial mesoderm (*p* = 1.4 × 10^–4^) (Supplemental Fig. 1B).

### Genetic mutations and embryological origins of the meninges

**Figure 2:**
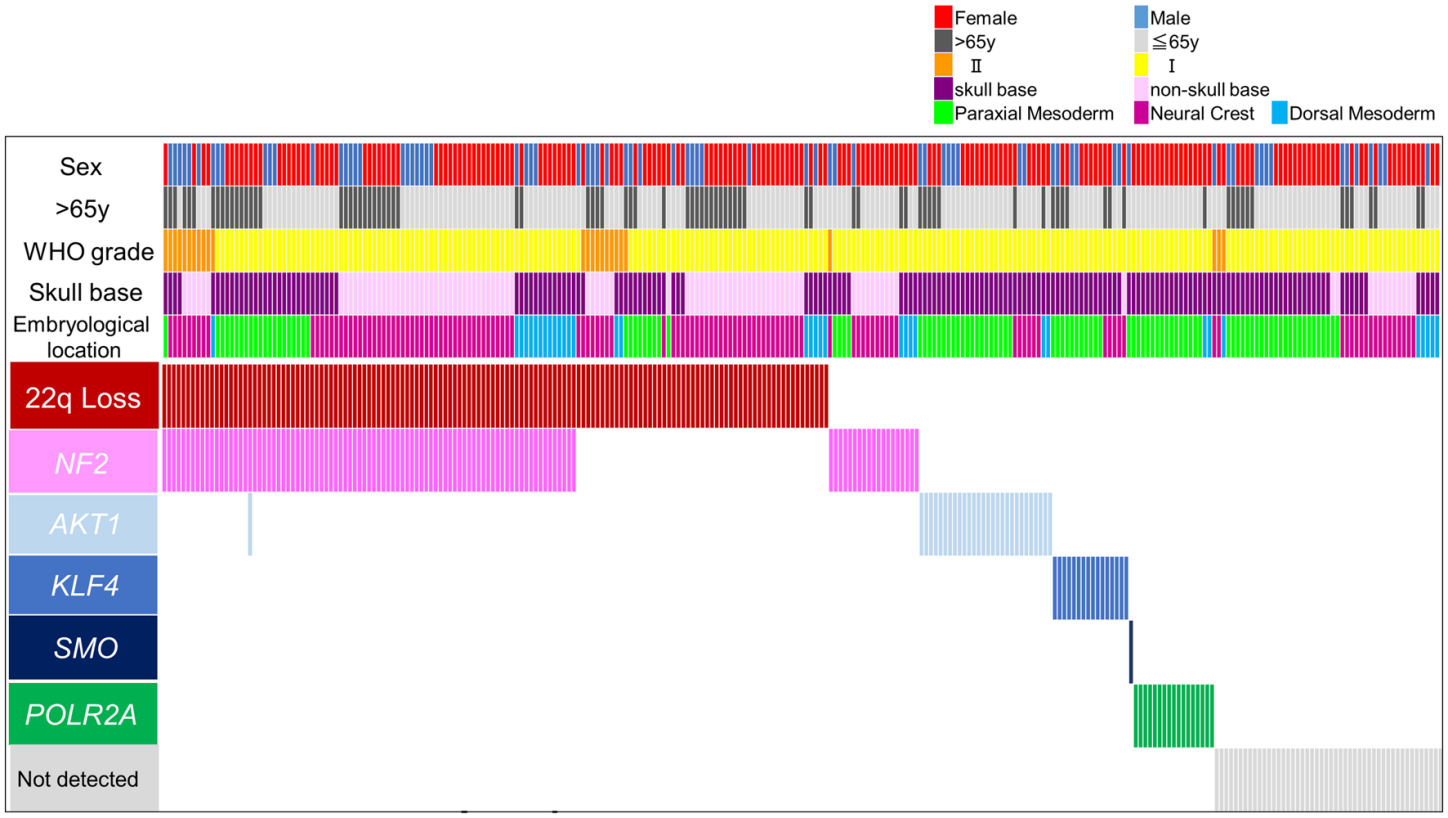
Overall genetic status and clinical characteristics. *NF2* mutation and/or 22q loss, *AKT1*, *KLF4*, *POLR2A* and *SMO* mutation are mutually exclusive and only one tumor harbored *NF2* and *AKT1* mutations. The remaining 48 cases were defined as “Not detected,” implying that none of these mutations were detected. 269 cases are depicted. Figure generated using Microsoft PowerPoint 2016, https://www.microsoft.com.

Mutations detected in the 269 cases were localized at *AKT1* in 29 cases, *KLF4* in 16 cases, *SM*O in one case, *POLR2A* in 17 cases*,* with *NF2* and 22q loss in 87 cases, and *NF2* only in 19 cases and 22q loss only in 53 cases (Fig. 2). Representative cases and the results of Sanger sequencing of each mutation are illustrated in Supplemental Fig. 2. These mutations were mutually exclusive and only one tumor harbored *NF2* and *AKT1* mutations. The remaining 48 cases were defined as “Not detected,” implying that none of these mutations were detected (Fig. 2).

**Figure 3:**
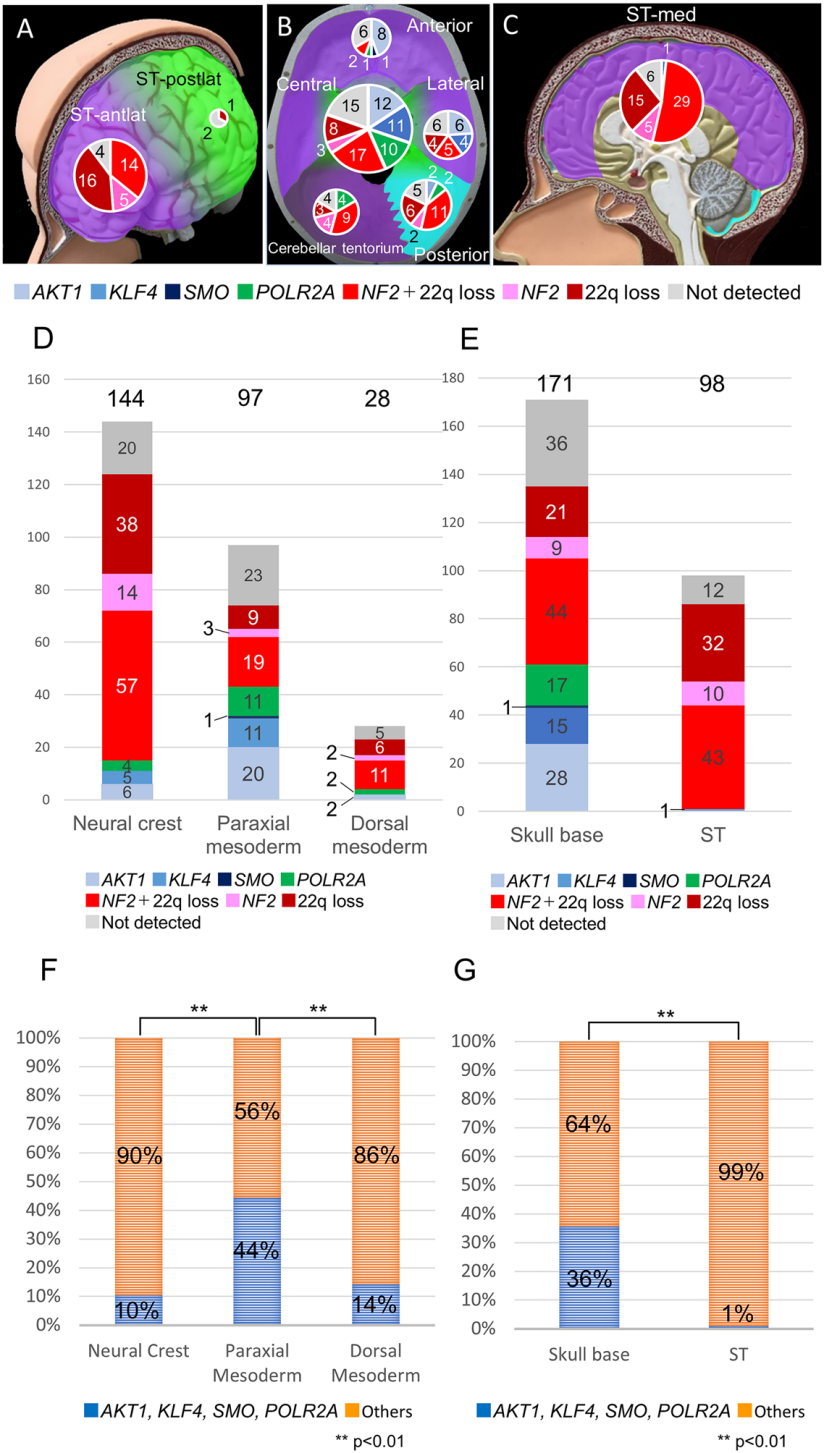
Genetic mutations according to embryologically classified anatomical location of meningiomas. (**A**) Distribution of genetic mutations at ST-ant/lat and ST-post/lat. Each pie chart shows the breakdown of the number of patients harboring each type of mutation. The sizes of the pie charts reflect the total number of the patients with each type of lesion. (**B**) Distribution of mutations in skull-base tumors. The numbers indicate the number of patients harboring tumors at each location. The sizes of the pie chart indicate the total number of patients with tumors at different locations. (**C**) Distribution of mutations at ST-med. The number indicates the number of patients with tumors at each location. The sizes of the pie chart indicate the total number of patients harboring tumors at each location. (**D**) The number of patients harboring mutations at each embryologically classified location. (**E**) The number of patients harboring tumors at the skull base or Supra tentorial area, with mutations. The bar graph shows the numbers of patients with each type of mutation per location. (**F**) Comparison of the ratio of patients harboring *AKT1*, *KLF4*, *SMO*, or *POLR2A* mutations among embryologically classified locations. The ratio of patients harboring *AKT1*, *KLF4*, *SMO*, or *POLR2A* mutations was higher for tumors originating from the paraxial mesoderm location rather than the neural crest or dorsal mesoderm (*p* < 0.01, significant on the chi-square test with Bonferroni correction). (**G**) Comparison of the ratio of patients harboring *AKT1*, *KLF4*, *SMO*, or *POLR2A* mutations between skull-base and supra-tentorial tumors. The ratio of patients harboring *AKT1*, *KLF4*, *SMO*, or *POLR2A* mutations was higher for skull-base tumors rather than supra tentorial tumors (*p* < 0.01, significant on the chi-square test with Bonferroni correction). ST-ant/lat, supra-tentorial-antero-lateral; ST-post/lat, supra-tentorial-postero-lateral; ST-med, supra-tentorial-medial. Figure generated using Microsoft PowerPoint 2016, https://www.microsoft.com.

Tumors in almost all patients harboring any one of these four mutations were present along the skull base, with the exception of one patient harboring a *KLF4* mutation wherein the tumor was located in the ST-post/lat region. Notably, only one meningioma with an *SMO* mutation had an anterior location; however, numerous tumors harboring *KLF* or *POLR2A* mutations had a central location. Furthermore, numerous tumors harboring an *AKT1* mutation had anterior and central locations (Fig. 3A–C).

Regarding the association between genetic alterations and embryological origins of the meninges, Fig. 3D shows the numbers of patients harboring each genetic mutation at each embryologically classified region. Figure 3E shows the numbers of patients harboring each genetic mutation at the skull base or supra tentorial. In particular, *AKT1, KLF4, SMO*, or *POLR2A* mutations were significantly more frequent in meningiomas of paraxial mesodermal origin than in those of neural crest (*p* = 1.7 × 10^–10^) and dorsal mesodermal origin (*p* = 3.0 × 10^–4^) (Fig. 3F). *AKT1, KLF4, SMO*, or *POLR2A* mutations were significantly more frequent in meningiomas of skull-base lesions than in those of supra-tentorial lesions (*p* = 8.3 × 10^–11^) (Fig. 3G). Regarding patients harboring *NF2* mutations and/or 22q loss, these mutations were more frequent in meningiomas of neural crest than in those of paraxial mesodermal origin (*p* = 3.9 × 10^–12^) and more frequent in those of neural crest rather than dorsal mesodermal origin (*p* = 5.0 × 10^–5^) (Supplemental Fig. 3).

### Genetic mutations and pathological diagnosis

**Figure 4:**
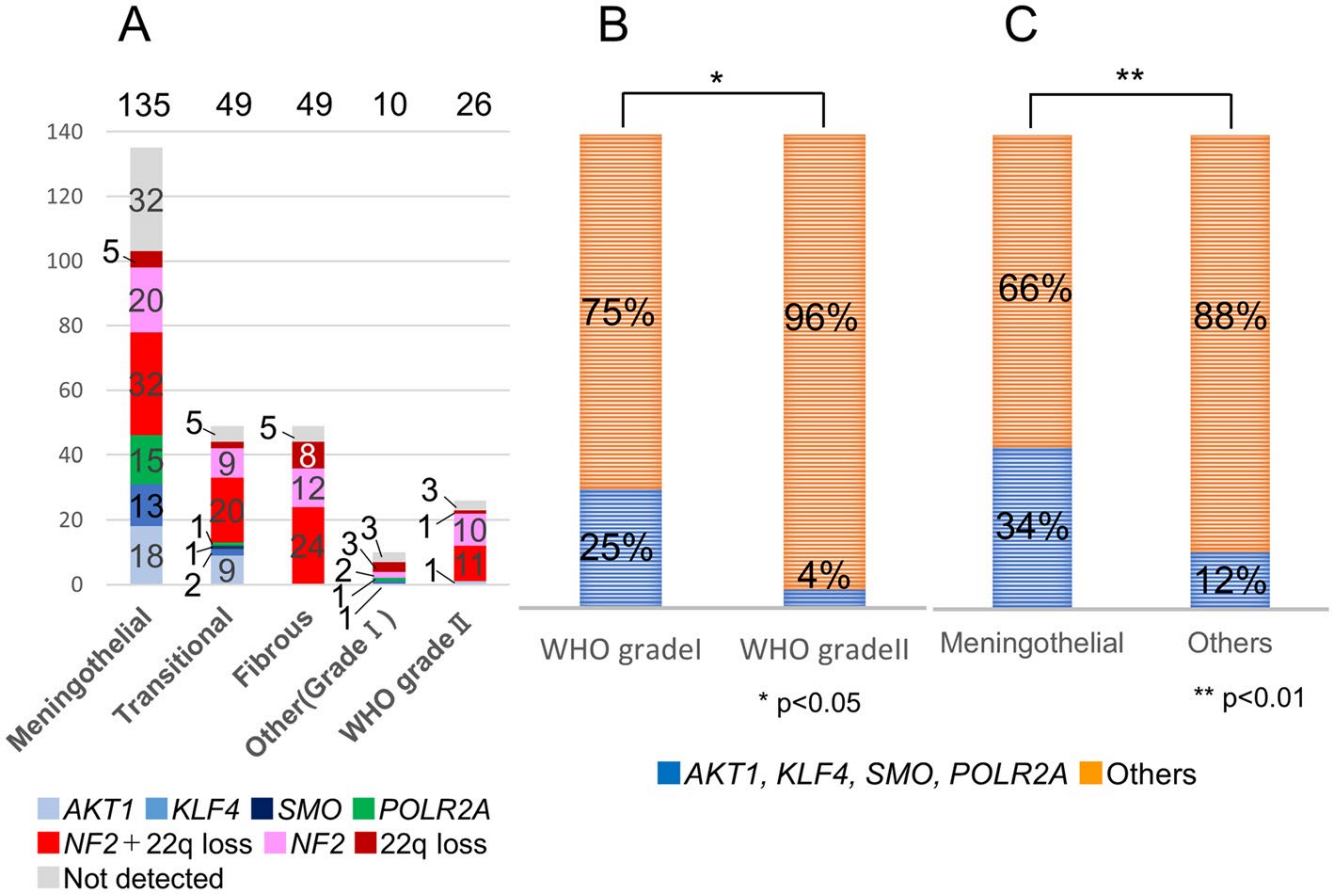
Distribution of genetic mutations among different pathological diagnosis. (**A**) The number of patients harboring mutations for each pathological diagnosis. (**B**) Comparison of the ratio of patients harboring *AKT1*, *KLF4*, *SMO*, or *POLR2A* mutations between WHO grade I and WHO grade II tumors. The ratio of patients harboring *AKT1*, *KLF4*, *SMO*, or *POLR2A* mutations was significantly higher for WHO grade I than for WHO grade II tumors (*p* < 0.05, significant on the chi-square test with Bonferroni correction). (**C**) Comparison of the ratio of patients harboring *AKT1*, *KLF4*, *SMO*, or *POLR2A* mutations between meningothelial meningioma and other pathologies. The ratio of patients harboring *AKT1*, *KLF4*, *SMO*, or *POLR2A* mutations was higher for meningothelial meningioma than for other pathologies (*p* < 0.01, significant on the chi-square test with Bonferroni correction).

The number of patients harboring each mutation based on pathological diagnosis is indicated in a bar graph in Fig. 4A. *AKT1, KLF4, SMO*, or *POLR2A* mutation was significantly more frequent in WHO grade I meningioma than in WHO grade II meningioma (*p* = 0.01) (Fig. 4B), and any one of these four mutations was more frequently associated with meningothelial histological types than with other pathological types. (*p* = 1.6 × 10^–5^) (Fig. 4C). As an exception, one patient presented with a psammomatous meningioma with a *POLR2A* mutation (Supplemental Fig. 4), and another patient presented with an angiomatous meningioma with a *KLF4* mutation, while yet another patient presented with an atypical meningioma with an *AKT1* mutation. However, the 49 patients with fibrous and 25 patients with WHO grade II meningiomas did not harbor these four mutations, except one patient. Forty-four of the 49 patients (89.8%) with fibrous type and 22 of the 25 patients (88.0%) with WHO grade II meningiomas harbored an *NF2* mutation or 22q loss. Among WHO grade I tumors, *NF2* mutations or 22q loss were significantly more frequent in fibrous meningioma than in other pathological types (*p* = 1.3 × 10^–7^) (Supplemental Fig. 5).

**Figure 5:**
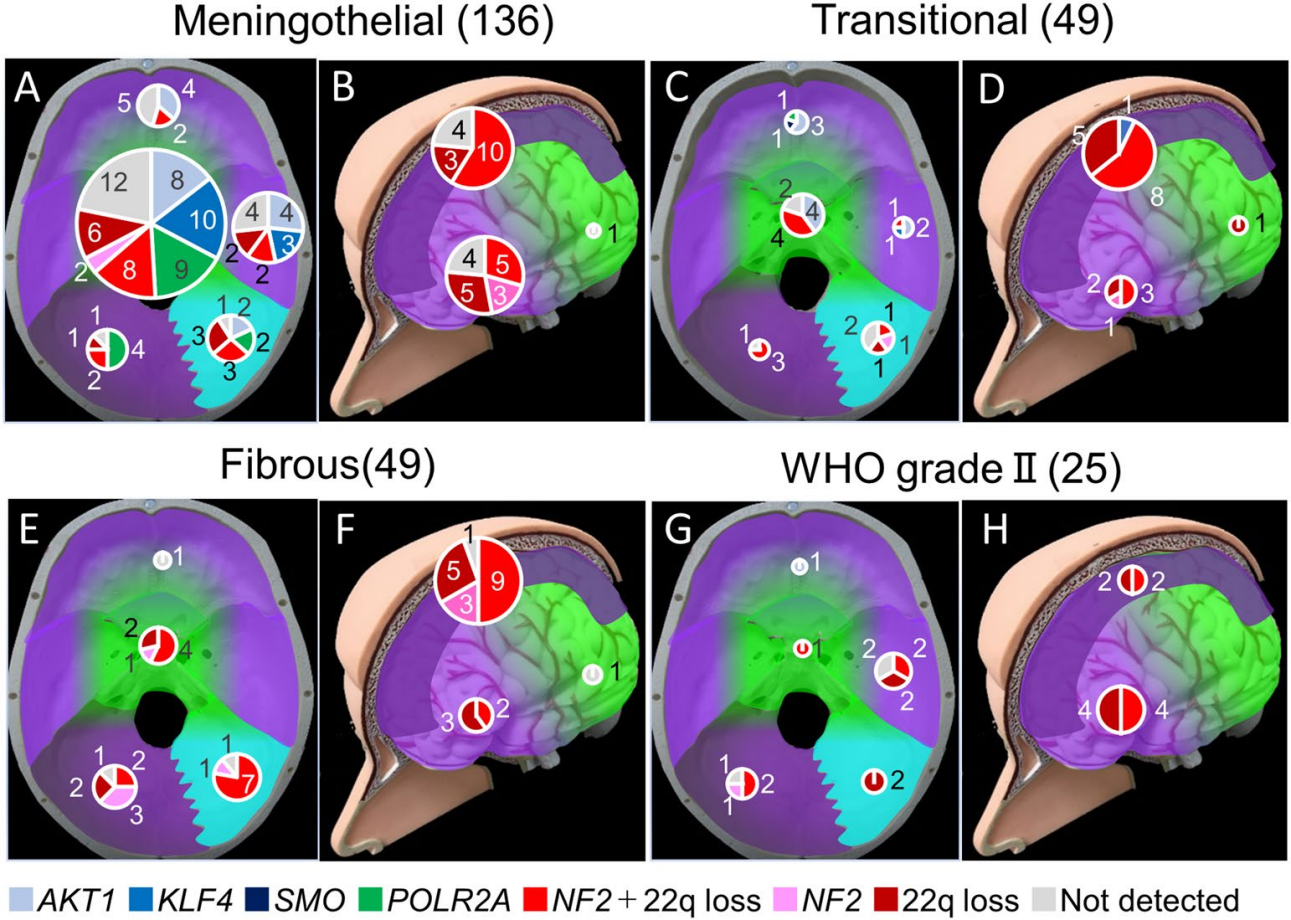
Associations between genetic mutations, histological type, and tumor locations. Each pie chart shows the breakdown of patients harboring each type of mutation. The size of pie charts reflects the total number of patients harboring each type of lesion. (**A**) Meningothelial tumor in the skull base. (**B**) Meningothelial tumor in the supra tentorial region. (**C**) Transitional tumor in the skull base. (**D**) Transitional tumor in the supra tentorial region. (**E**) Fibrous tumor in the skull base. (**F**) Fibrous tumor in the supra tentorial region. (**G**) WHO grade II tumor in the skull base. (**H**) WHO grade II tumor in the supra tentorial region. Figure generated using Microsoft PowerPoint 2016, https://www.microsoft.com.

Figure 5 summarizes the anatomogenetic characteristics of each type of pathology.

### Effects on the recurrence rates of meningioma

**Figure 6:**
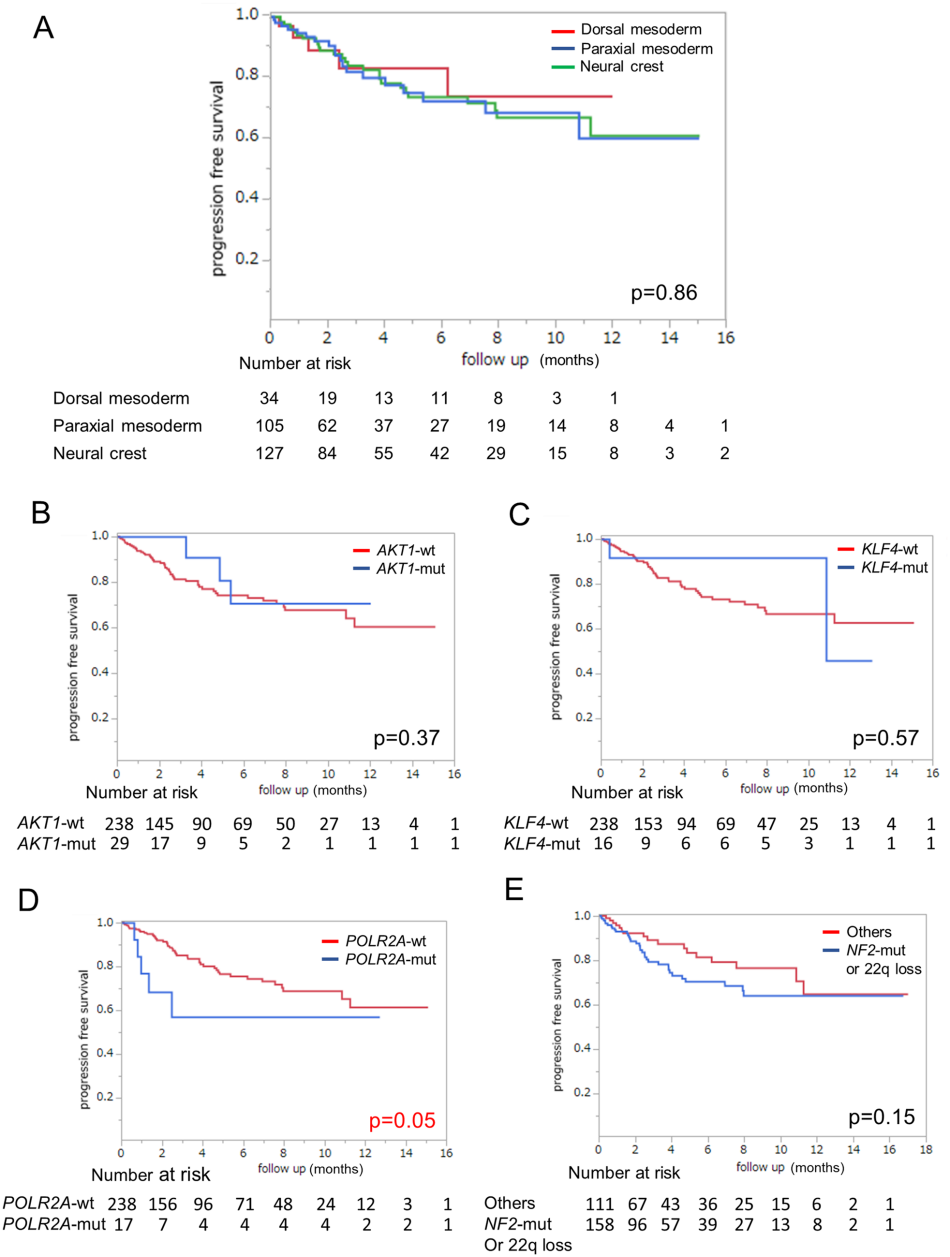
Kaplan–Meier plots of time to recurrence based on genetic mutations. (**A**) Comparison among tumors originating from the dorsal mesoderm, paraxial mesoderm, and neural crest. No significant differences among the three groups (*p* = 0.86). (**B**) Comparison between *AKT1* mutant and *AKT1* wild type. No significant difference between the two groups (*p* = 0.37). (**C**) Comparison between *KLF4* mutant and *KLF4* wild type. No significant difference between the two groups (*p* = 0.57). (**D**) Comparison between *POLR2A* mutant and *POLR2A* wild type. Progression-free survival was significantly worse among patients harboring mutant than wild type *POLR2A* (*p* = 0.05). (**E**) Comparison between *NF2* mutant or 22q loss and other mutation types. No significant difference between the two groups (*p* = 0.15).

In this study, embryologically classified tumor locations were not prognostic factors both with the log-rank test (*p* = 0.86) (Fig. 6A) and a Cox proportional hazards model (Table 2). However, Kaplan–Meier curves comparing tumor recurrence among patients harboring different mutations and those without these mutations revealed that the presence of a mutation may potentially play a predictive role (Fig. 6B–E). Patients harboring *POLR2A* mutations experienced tumor recurrence with a high rate of 29.4% (Table 1). This group displayed a significant difference on the log-rank test (*p* = 0.05) (Fig. 6D). Furthermore, we analyzed the factors associated with tumor recurrence through a Cox proportional hazards model (Table 2). Multivariate analysis was performed using factors with a *p *value of ≤ 0.20 on univariate analysis. In the multivariate model, WHO grade II (*p* = 1.2 × 10^–4^, Hazard Ratio [HR] 4.99, 95% Confidence Interval [CI] 2.20–11.3), Simpson grade 1–3 (*p* = 1.9 × 10^–6^, HR 0.21, 95% CI 0.11–0.39), and *POLR2A* mutation (*p* = 1.7 × 10^–2^, HR 4.08, 95% CI 1.28–13.0) were associated with tumor recurrence (Table 2).

**Table 2:** Risk factors for tumor recurrence.

	Univariate			Multivariate		
	Hazard ratio	*p* value	95% CI	Hazard ratio	*p* value	95% CI
Female	0.76	0.37	0.42–1.38			
Age ≥ 65	1.49	0.21	0.80–2.78			
Simpson grade 1–3	0.16	9.7 × 10^–10^	0.09–0.29	0.21	1.9 × 10^–6^	0.11–0.39
MIB-1 LI ≥ 3	3.33	1.1 × 10^–4^	1.81–6.14	1.81	0.14	0.82–3.98
Meningothelial	0.72	0.24	0.41–1.25			
Transitional	0.96	0.92	0.43–2.14			
Fibrous	0.47	0.11	0.19–1.19	1.00	0.99	0.32–3.14
WHO grade II	6.44	3.9 × 10^–9^	3.46–12.0	4.99	1.2 × 10^–4^	2.20–11.3
Neural crest	1.00	0.99	0.58–1.75			
Paraxial mesoderm	1.07	0.81	0.61–1.90			
Dorsal mesoderm	0.83	0.70	0.33–2.10			
*AKT1* mutation	0.60	0.39	0.19–1.94			
*KLF4* mutation	0.67	0.55	0.11–2.15			
*POLR2A* mutation	2.42	0.09	0.83–5.58	4.08	1.7 × 10^–2^	1.28–13.0
*NF2* mutation or 22q loss	1.48	0.18	0.84–2.72	1.81	0.15	0.81–4.04

*LI* indicates labelling index, *ST-med* supra-tentorial-medial, *ST-ant/lat* supra-tentorial-antero-lateral, *ST-post/lat* supra-tentorial-postero-lateral, *CI* confidence interval.

Five of 17 patients harboring a *POLR2A* mutation experienced tumor recurrence. All five patients underwent partial tumor resection during initial surgery because the tumor was located at the central skull base. Nevertheless, the presence of the *POLR2A* mutation served as a determinant of tumor recurrence independent of Simpson grade 1–3. The follow-up duration among patients harboring the *POLR2A* mutation did not markedly differ from that of patients harboring other mutations. Furthermore, all patients harboring the *POLR2A* mutation were followed-up at our institution. We examined the effect of the *POLR2A* mutation among skull-base WHO grade I tumors and found that patients harboring the *POLR2A* mutation had a significantly worse prognosis (*p* = 8.9 × 10^–3^) (Supplemental Fig. 6). Furthermore, we analyzed the factors associated with tumor recurrence through a Cox proportional hazards model in this group (Supplemental Table 1). Multivariate analysis was performed using factors with a p-value of ≤ 0.20 on univariate analysis. In the multivariate model, Simpson grade 1–3 (*p* = 1.5 × 10^–3^, HR 0.25, 95% CI 0.11–0.59), MIB-1 LI** ≥ **3 (*p* = 0.01, HR 3.26, 95% CI 1.27–8.40) and *POLR2A* mutation (*p* = 0.04, HR 2.80, 95% CI 1.16–9.53) were significantly associated with tumor recurrence. Hence, the *POLR2A* mutation may be a potentially useful predictor of tumor recurrence in skull-base WHO grade I meningiomas.

## Discussion

This study comprehensively illustrates the anatomopathological association among driver mutations in meningiomas. Associations among anatomical locations, pathological diagnosis, and driver mutations were consistent with those reported previously^12,16,19^. However, this study reveals novel findings about the associations of the embryological origins of the meninges at different anatomical locations with genetic backgrounds and pathological diagnoses. Moreover, this study reports that the *POLR2A* mutation may serve as a potential marker for meningiomas with poor prognoses.

This study defined the locations of tumor origins on the basis of existing knowledge of the embryological origin of leptomeninges. Meningeal convexities originate from the skeleto-genesis layer immediately adjacent to them^20^. Furthermore, the calvaria develops from the embryonic head mesenchyme surrounding the brain, similar to the meninges; hence, its progenitors are presumably included in the primary meninx^21^, suggesting a similar origin of the meninges and bones. In mammals, the skull vault is constructed from embryogenic tissues of the neural crest and mesoderm^22,23^. The coronal suture separates the neural-crest-derived frontal bone from the paraxial mesodermal parietal bone^22,25^. Therefore, the anterior region of the meningeal convexity is expected to originate from the neural crest and the posterior region from the paraxial mesoderm.

Falx cerebri and cerebellar tentorium are derived from the neural crest^20,22^. Differences in the prechordal plate differentiates into the neural crest and forms the cerebellar tentorium adjacent it^28^. The neural crest forms the anterior region of the falx. After the posterior region of the falx is formed, both regions interact.

Regarding skull-base meninges, McBratney-Owen et al. reported that in rats, the anterior cranial base is derived from the neural crest, the posterior cranial base is derived from the mesoderm and the sphenoid bone is largely derived from the neural crest^24^. However, the border line of these embryological contributions is different depending on the species investigated^23^. Overall, these studies indicated that the central skull base is derived from the paraxial mesoderm and the posterior skull base originates from the dorsal mesoderm. The lateral region, including the sphenoid wing, is derived from the neural crest and the anterior skull base does not have a clear border; however, a narrower transitional zone between paraxial mesoderm and neural crest is formed^23,24^.

We evaluated the association between tumor location and pathological diagnoses by defining locations in accordance with the embryological origin of the meninges. The tumors in the neural crest originating regions were associated with more frequent fibrous and WHO grade II meningiomas. However, the tumors in the paraxial mesoderm originating locations were associated with more frequent meningothelial meningioma. It was difficult to evaluate meningiomas originating from the dorsal mesoderm owing to the small patient cohort herein; however, tendencies were similar to those of neural crest origin area. The association between tumor location and histopathology might be based on embryonic dural development. Further studies are required to prove this hypothesis.

This study showed the association between genetic alterations and tumor locations consistent with the previous reports^8,12,15,29^. From the embryological viewpoint, we found that the meningiomas harboring *AKT1, SMO, KLF4,* or *POLR2A* mutations were significantly associated with paraxial mesodermal origin. However, meningiomas with *NF2* mutations or 22q loss were significantly associated with neural crest and dorsal mesoderm origin.

Previous study have reported that sensitivity to *NF2* loss-of-function mutations differ between mesoderm-derived and neural crest-derived meninges in transgenic mice^30^. Recently, Boetto et al.^26^ reported that selective sensitivity of the skull-base arachnoid to *SMO* activation initiated meningothelial meningioma in the transgenic mice. These results may explain the differences in genetic status and pathology in accordance with tumor location. However, further biological studies on humans are necessary.

This study indicates associations between driver mutations and histological findings, consistent with previous reports^12,15^. The proportion of meningothelial meningiomas harboring *AKT1, KLF4, SMO*, or *POLR2A* mutations was significantly high. Fibrous and WHO grade II tumors primarily harbored an *NF2* mutation or 22q loss.

This study, along with previous studies, clearly suggests that among WHO grade I meningiomas, the genetic background of the fibrous type differed from that of meningothelial and transitional types^12,29^. Previous studies have reported that in a transgenic mouse, meningothelial meningiomas originated from arachnoid barrier cells and fibrous meningiomas originated from dural border cells^26,30^. Apparently, these cells differ in their sensitivity to specific genetic mutations, depending on the tumor location^26,30,31^. These results further corroborate the present association between pathological diagnoses and mutations.

This study shows that the *POLR2A* mutation is a potentially suitable marker for meningiomas with poor prognoses, especially among WHO grade I skull-base meningiomas. Furthermore, the *POLR2A* mutation was most frequently observed in the central region, which is originated from the paraxial mesoderm. *POLR2A* is located at 17p13.1 and encodes RNA polymerase II, which plays a fundamental role in eukaryotic organisms. The detailed biological role of the *POLR2A* mutation in meningiomas remains currently unknown^32^. An RNA polymerase inhibitor, alpha-amanitin, is reported to suppress colorectal tumors harboring the *POLR2A* mutation^33^. Alpha-amanitin could be a candidate for molecular targeted therapy for the meningiomas harboring *POLR2A* mutation. The clinical and biological characteristics of meningiomas harboring *POLR2A* mutation warrant further clarification.

One limitation of our study is its retrospective, single-center design. Another limitation would be that we analyzed a limited number of genetic mutations. Mutations in the genes analyzed herein are frequent; however, other mutations including those in *TRAF7*, *hTERT*, *SMARCB1*, *SUFU*, and *PIK3*CA also occur in meningiomas^15,34–37^. Moreover, tumorigenesis in meningiomas is associated with not only these driver genetic mutations but also global gene expression profiles and methylation status^38^. This study analyzed genes with hotspot point mutations through Sanger Sequencing and 22q loss through microsatellite analysis. We speculate that these genes can be easily analyzed in the clinical setting. To completely elucidate the association between meningioma tumorigenesis and the embryological origin of the meninges, comprehensive genetic analysis including that of global expression profiles and methylation status is necessary.

## Conclusion

This study shows that meningiomas, according to the embryologic origin of their dural attachment, display differences in pathological diagnosis and genetic abnormalities. Furthermore, this study is the first to show that the *POLR2A* mutation is a potential indicator of increased tumor recurrence. Assessment of the embryological origin of the meninges may provide novel insights into the pathomechanism of meningiomas. Future molecular biological studies on meningeal embryology are necessary.

## Materials and methods

All methods were carried out in accordance with relevant guidelines and regulations.

### Patient population

This study was approved by the Institutional Review Board of the University of Tokyo (Approval Number G10028), and informed consent in writing was obtained from all subjects. We retrospectively analyzed data on 499 patients who underwent resection of meningiomas at the University of Tokyo Hospital between January 2000 and June 2017. We excluded 153 patients from whom fresh-frozen specimens or tumor DNA were not obtained. When the patient underwent multiple surgeries, data from only the first surgery were used. Sixty-eight patients having undergone previous tumor resection at another hospital were excluded. Furthermore, 2 patients harboring NF2, 2 patients having undergone radiation therapy before surgery, and 5 patients with multiple meningiomas were excluded from the study. Finally, the study included 269 patients.

### Data collection

We evaluated the following parameters: sex, age, location of origin (attachment to dura), pathological diagnosis, extent of resection (Simpson grade), the need for additional treatment (surgery or/and radiosurgery), and time to tumor recurrence by reviewing the clinical and surgical records. Location was initially defined in accordance with the existing surgical classification based on the anatomical location of tumor dural attachment, in order to accurately extract data from these records. Thus, we classified supra-tentorial locations on the convexity, falx, and parasagittal areas into three types: supra-tentorial-medial (ST-med), supra-tentorial-antero-lateral (ST-ant/lat), and supra-tentorial-postero-lateral (ST-post/lat). The border between the “anterior” and “posterior” convexities was the coronal suture. Furthermore, the skull-base lesions were classified into four locations: anterior, central, posterior, and lateral. “Anterior” lesions included the anterior cranial fossa, olfactory groove, and planum sphenoidale. “Central” lesions included the anterior clinoid process, posterior clinoid process, tuberculum sellae, Meckel’s cave, cavernous sinus, clival, petroclival-anterior to internal auditory meatus (IAM), and cerebellopontine angle (CPA)-anterior to IAM. “Lateral” lesions included the sphenoid wing and the tentorial attachments, extending into middle cranial fossa, and all middle fossa lesions. “Posterior” included the foramen magnum, CPA posterior to IAM, jugular foramen, cerebellar convexity, and tentorial—extending to the posterior fossa (Fig. 1). We classified the cases, which occupied broader areas, in accordance with the area of the most extensive attachment.

We defined the locations of tumor origin on the basis of the existing knowledge of the embryological origin of leptomeninges^20–22,24,27^. We generated a scheme of normal meningeal development in accordance with their origin (Supplemental Fig. 7). Furthermore, we defined the embryological origins of anatomical locations of the meninges as follows: neural crest origin, including “Lateral,” “ST-med,” “ST-ant/lat,” and “Cerebellar tentorium”; paraxial mesodermal origin, including the “Anterior,” “Central,” and “ST-post/lat”; dorsal mesodermal, including the “Posterior” group.

Patients were followed-up through contrast-enhanced magnetic resonance imaging (CE-MRI) at 2 d, 6 months, and 1 year after surgery. If there no tumor recurrence was observed, follow-up was regularly continued every year through CE-MRI. For MRI in all cases, we conducted a central review. The precise locations of the tumor origin were defined through preoperative MR images through inter-observer agreement between the neuro-radiologist and two neurosurgeons blinded to the clinical or genetic data. Furthermore, we defined tumor recurrence through inter-observer agreement between the neuro-radiologist and two neurosurgeons blinded to the clinical or genetic data, on the detection of apparent enlargement of residual tumors on CE-MRI.

We conducted a central review of all pathological diagnoses for cases in accordance with the 2016 WHO Classifications of Tumors of the Central Nervous System including the cases diagnosed in the basis of 2000 or 2007 WHO Classifications of Tumors of the Central Nervous System.Transitional meningioma was defined on the basis of the 2016 WHO Classifications of Tumors of the Central Nervous System as a WHO grade I meningioma characterized by the coexistence of meningothelial cells and fibrous architectural patterns. The MIB-1 LI was determined using the highest LI values in areas of their maximum density as identified through visual analysis.

### DNA extraction and Sanger sequencing

Tumor DNA was extracted from fresh-frozen tumors, using the QIAmp DNA minikit (QIAGEN; Venlo, Netherlands) in accordance with the manufacturer’s instructions, and DNA quality was evaluated using a spectrophotometer. We sequenced mutational hotspots of each gene except *NF2.* Mutations in *AKT1* (c.49G > A [p.Glu17Lys]), *KLF4* (c.1228A > C [p.Lys409Gln]), *SMO* (c.1234C > T [p.Leu412Phe] and c.1604G > T [p.Trp535Leu]), and *POLR2A* (c.1207C > A [p.Gln403Lys] or c.1310–1315 del ACCTTC [p.Leu438_His439del]) were screened through direct Sanger sequencing in all cases. The primers were designed using Primer3. Since *NF2* has no mutational hotspots, we performed direct Sanger sequencing for all exons, using the primers generated from the exon primer. For PCR, 50 ng of DNA and KOD FX NEO were used. PCR was performed with 20 µl reaction mixtures and the following reaction cycles: initial denaturation at 94 °C for 2 min, followed by 32 cycles with denaturation at 98 °C for 10 s, annealing at 58–60 °C for 30 s, and extension at 68 °C for 30 s, followed by final extension at 68 °C for 7 min. Sequences were determined using an ABI 3130xl Genetic Analyzer (Applied Biosystems).

### Microsatellite analysis

We performed microsatellite analysis to detect 22q Loss. This analysis aimed to compare germline and tumor DNA, using both blood and tumor samples. In our study, blood samples were obtained from 241 of the 269 patients. We used the following five microsatellite polymorphic markers franking *NF2*, selected from the Genome Data Base: *D22S268*, *D22S1163, D22S929, D22S280*, and *D22S282.* The sense primer was labeled with a fluorescent dye, and PCR was performed for 25–30 cycles at 58–60 °C for annealing, using the Gene Amp 9700 Thermal Cycler (PE Biosystems; Framingham, Massachusetts, USA). PCR products were separated through capillary electrophoresis with the Genetic Analyzer 310, and the analysis was performed using the Gene Scan Program (PE Biosystems)^39^.

### Statistical analysis

To determine the association between tumor location and pathological diagnosis and between tumor location and genetic mutational status, the chi-square test was used. For multiple comparisons, Bonferroni correction was applied. Progression-free survival was defined as the time between surgery and recurrence or final follow-up. Recurrence-free cases were censored on final follow-up. Kaplan–Meier survival curves were plotted and differences in progression-free survival between groups were compared using the log-rank test. We assessed the effect of sex, age, Simpson grade, MIB-1 LI, pathological diagnosis, embryological tumor localization, and mutational status through univariate analyses with a Cox proportional hazards model. Thereafter, multivariate analysis was performed using parameters with a *p* value of < 0.2 on univariate analysis. All statistical analyses were performed with JMP Pro version 11 (SAS Institute, Inc.; Cary, North Carolina, USA). A *p* value of < 0.05 was considered statistically significant. We excluded *SMO* mutation from the evaluating factor since there was only 1 case with *SMO* mutation.

## Supplementary Information

Supplementary Figures and Tables

## Data Availability

Data are available on reasonable request. The authors confirm that the data supporting the findings of this study will be shared by request from any qualified investigator.
