# Supplementary material for: Associations of pathological diagnosis and genetic abnormalities in meningiomas with the embryological origins of the meninges
Source: Sci Rep. 2021 Mar 26;11:6987. doi: 10.1038/s41598-021-86298-9 (PMC7998008; doi:10.1038/s41598-021-86298-9)
Supplement: Supplementary file 1 — Supplementary Figures and Tables [file 41598_2021_86298_MOESM1_ESM.doc]

Associations of pathological diagnosis and genetic abnormalities in meningiomas with the embryological origins of the meninges

Atsushi Okano^1^, M.D., Ph.D., Satoru Miyawaki^1^, M.D., Ph.D., Hiroki Hongo^1^, M.D., Shogo Dofuku^1^, M.D., Yu Teranishi^1^, M.D., Ph.D., Jun Mitsui^2^, M.D., PhD., Michihiro Tanaka^3^, M.D., PhD., Masahiro Shin^1^, M.D., Ph.D., Hirofumi Nakatomi^1^, M.D., Ph.D., Nobuhito Saito^1^, M.D., Ph.D.

1 Department of Neurosurgery, Faculty of Medicine, The University of Tokyo

Address: 7-3-1 Hongo, Bunkyo-ku, Tokyo, Japan

2 Department of Molecular Neurology, Graduate School of Medicine, The University of Tokyo Address: 7-3-1 Hongo Bunkyo-ku, Tokyo, Japan

3 Departments of Neurosurgery, Kameda Medical Center

Address: 929 Higashi-cho, Kamogawa, Chiba

**Supplemental File**

Supplemental figure 1


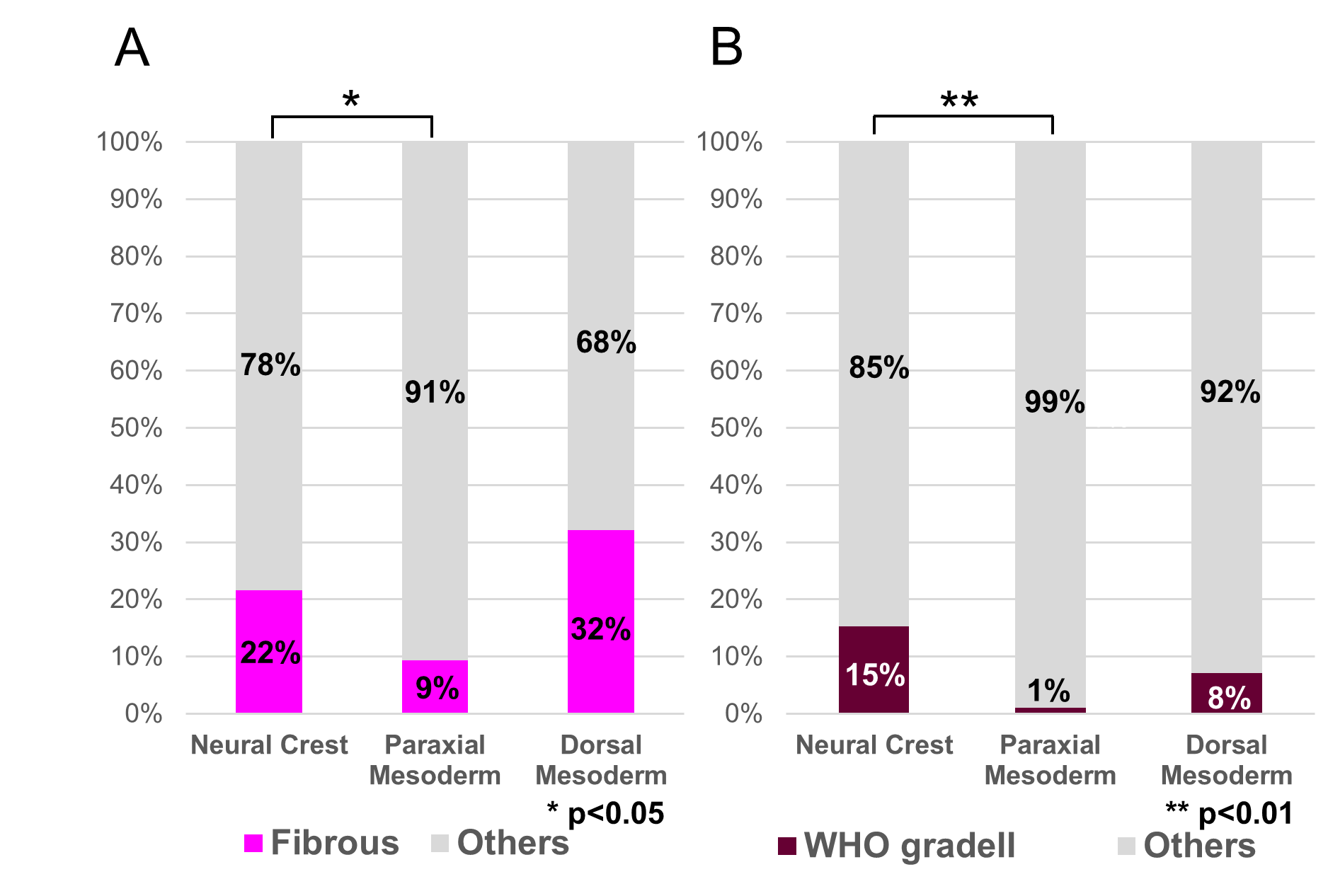


Supplemental Figure 1 Comparison of the ratio of pathological diagnoses between tumors of different embryological origin.

A: Comparison of the ratio of fibrous meningiomas and other pathologies at locations associated with different embryological origins. The ratio of fibrous meningiomas originating from the neural crest location was higher than those originating from the paraxial mesoderm (p<0.05, significant on the chi-square test with Bonferroni correction)

B: Comparison of the ratio of WHO grade II meningiomas and other pathologies at locations associated with embryological origin. The ratio of WHO grade II meningiomas in the neural crest was higher than those originating in the paraxial mesoderm (p<0.01, significant on the chi-square test with Bonferroni correction)

Supplemental Figure 2


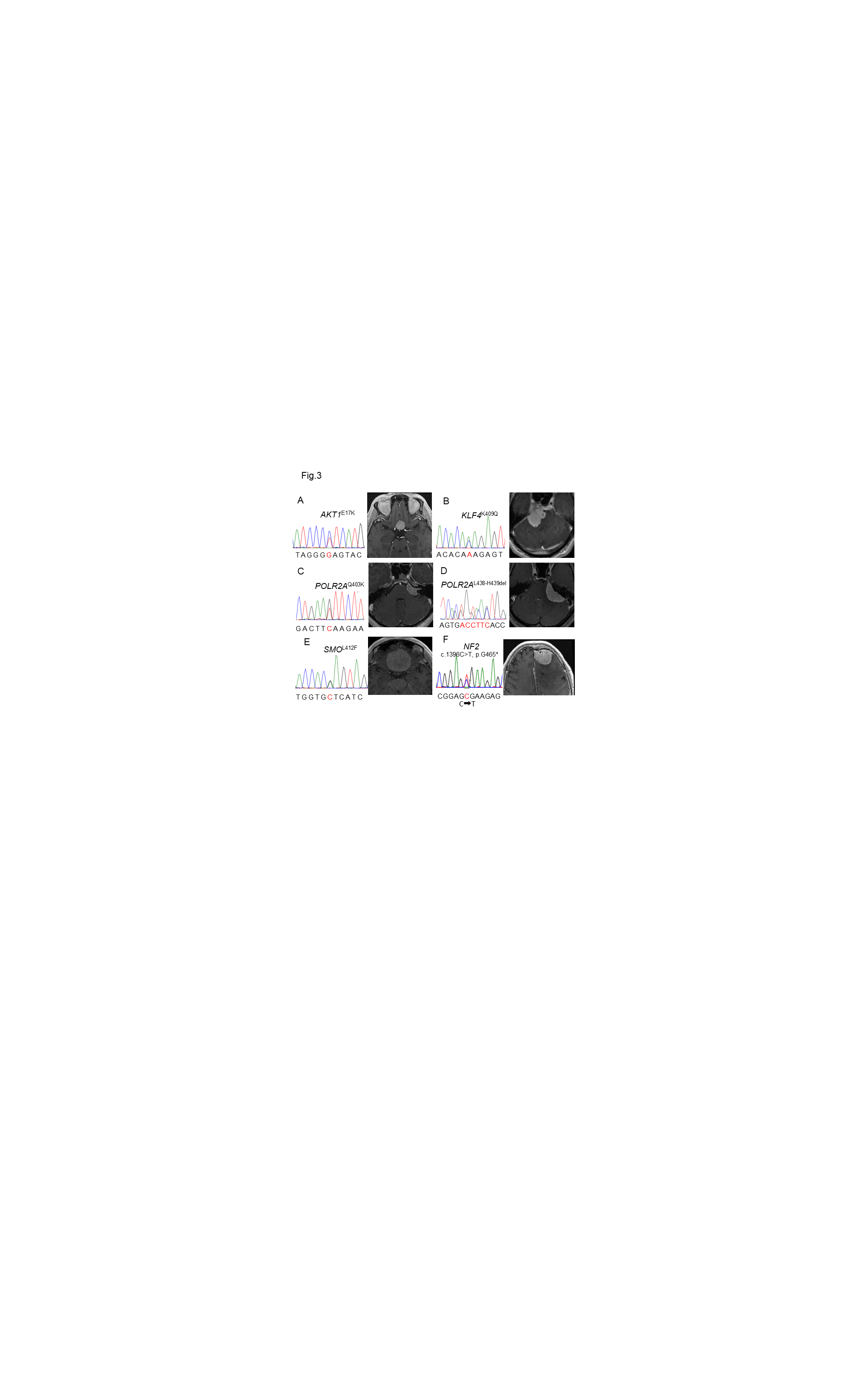


Supplemental Figure 2 Results of Sanger sequencing and representative images of patients harboring *AKT1*, *KLF4*, *POLR2A*, *SMO*, and *NF2* mutations

A: Results of Sanger sequencing of a patient harboring an *AKT1*^E17K^ mutation. This is a case of tuberculum sellae meningioma.

B: Results of Sanger sequencing of a patient harboring a *KLF4*^K409Q^ mutation. This is a case of petroclival meningioma.

C: Results of Sanger sequencing of a patient harboring a *POLR2A*^Q403K^ mutation.　This is a case of petrous meningioma.

D: Results of Sanger sequencing of a patient harboring a *POLR2A*^L438-H439^ deletion. This is a case of petrous meningioma.

E: Results of Sanger sequencing of a patient harboring an *SMO*^L412F^ mutation. This is a case of olfactory groove meningioma.

F: Results of Sanger sequencing of a patient harboring an *NF2* mutation. This is a case of convexity meningioma representing occlusion of the left middle cerebral artery.

Supplemental figure 3


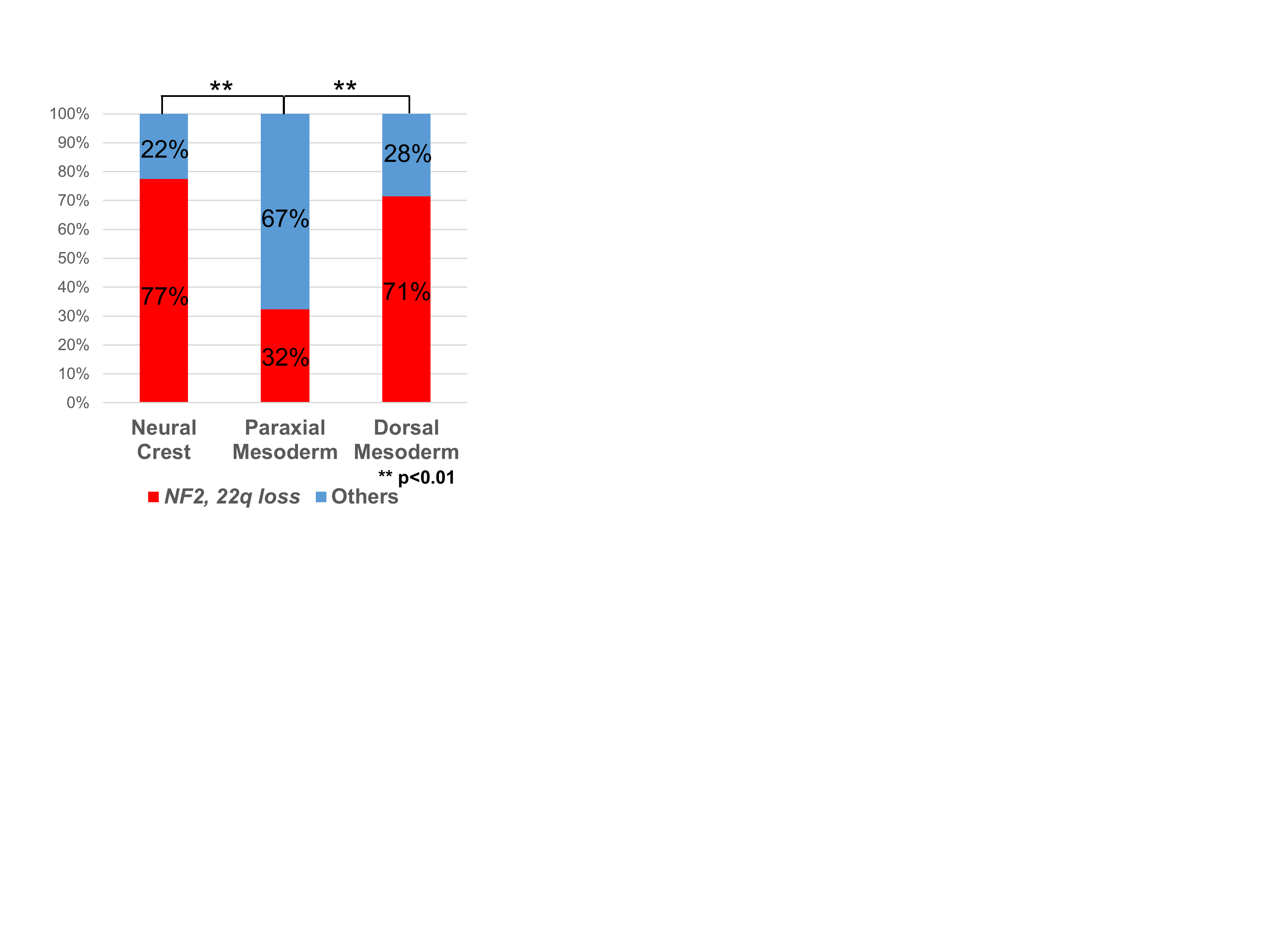


Supplemental Figure 3 Comparison of patients harboring an *NF2* mutation or 22q loss between embryologically classified locations

The ratio of patients harboring an *NF2* mutation or 22q loss was lower in tumors originating from the paraxial mesoderm rather than the neural crest or dorsal mesoderm (p<0.01, significant on the chi-square test with Bonferroni correction)

Supplemental figure 4


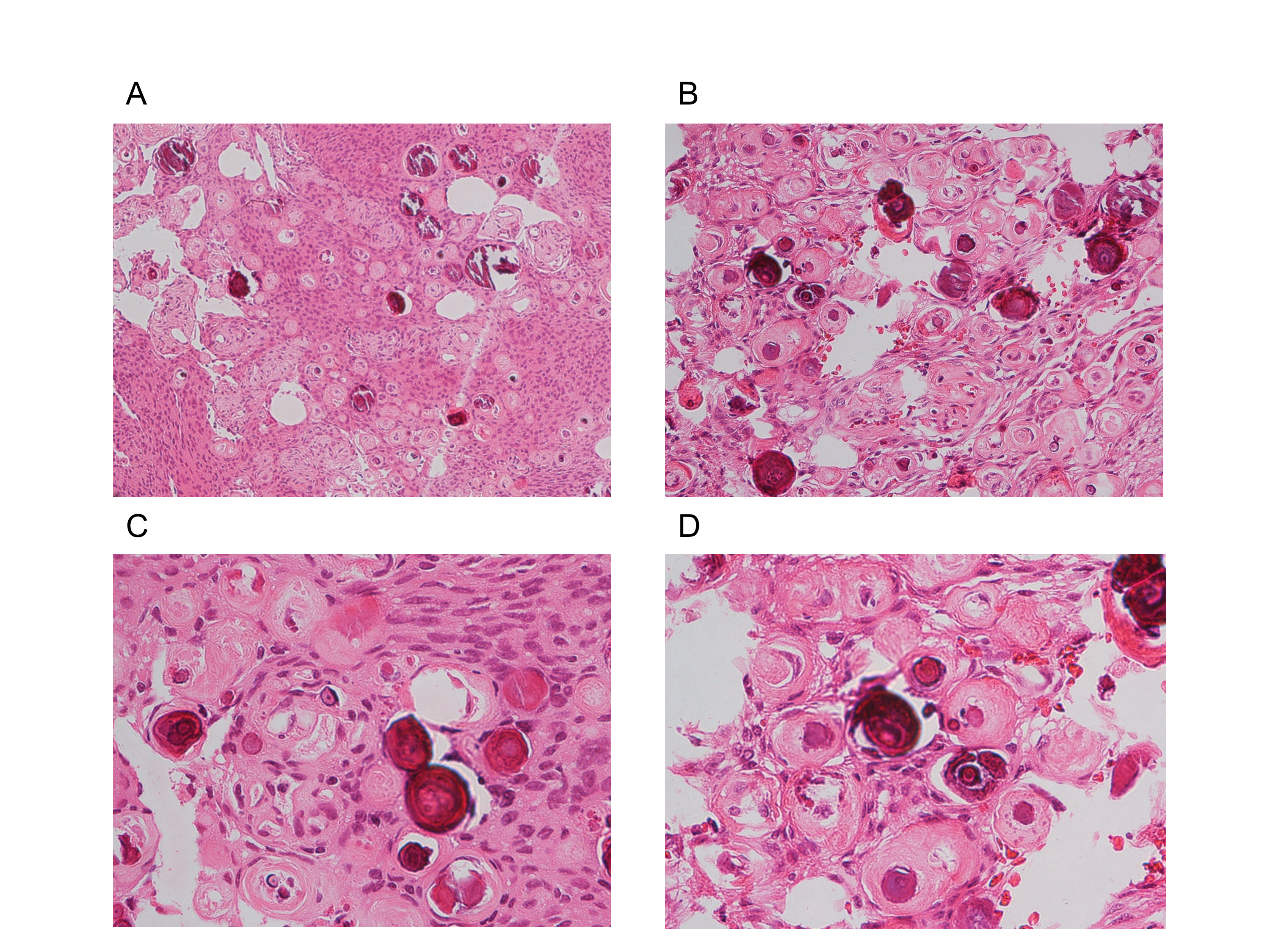


Supplemental Figure 4 Pathology of psammomatous meningioma in a patient harboring the *POLR2A* mutation

A, B: Uniform proliferation of spindle cells and numerous psammomatous bodies (hematoxylin-eosin [HE] staining: magnification, ×10, ×20)

C, D: Psammomatous bodies and whorl patterns. Psammomatous bodies show concentric calcification (HE staining: magnification, ×40)

Supplemental Figure 5


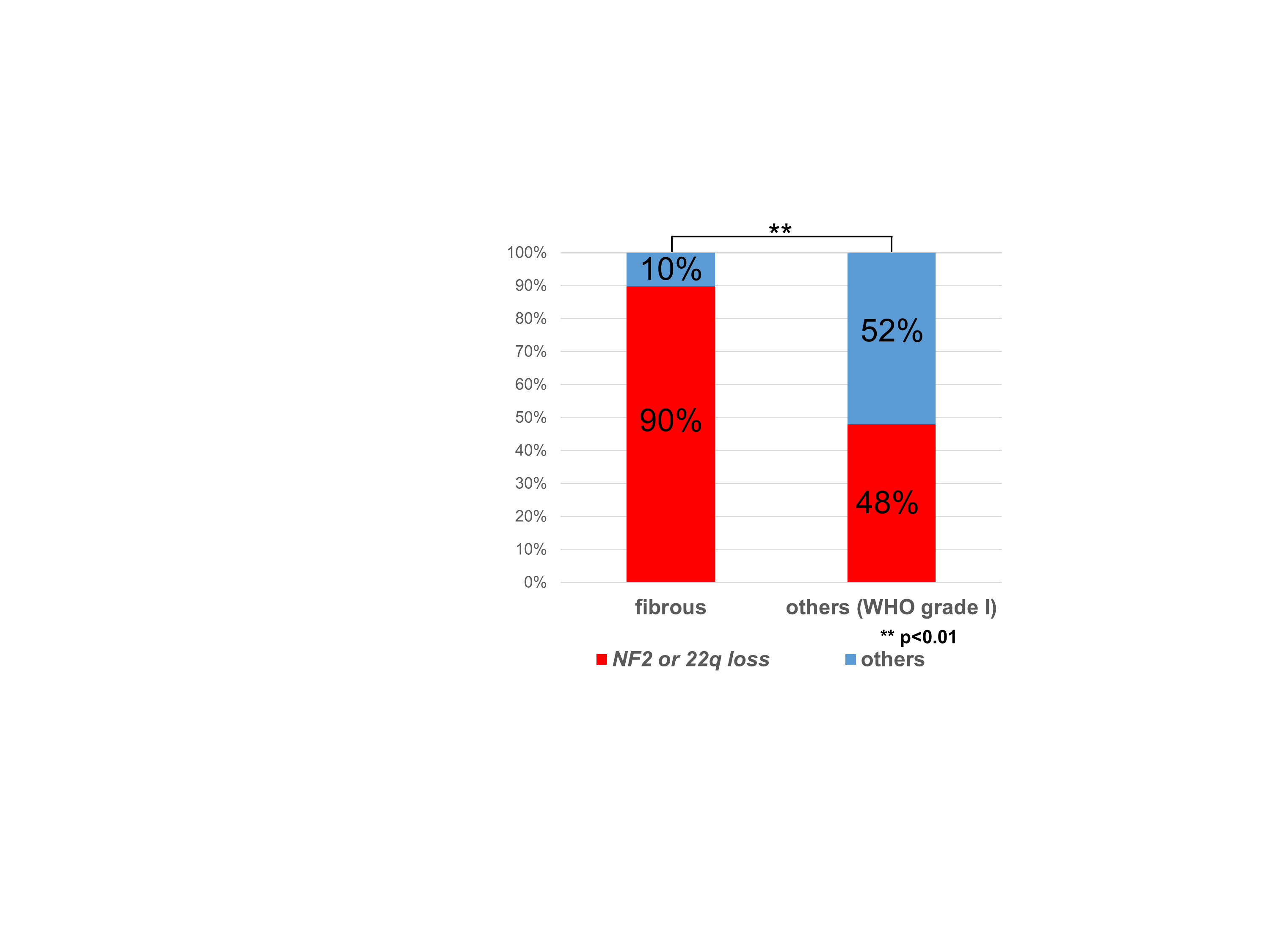


Supplemental Figure 5 Comparison of the ratio of patients harboring *NF2* mutation or 22q loss between fibrous type and other pathological types (WHO grade I)

The ratio of patients harboring an *NF2* mutation or 22q loss was higher in fibrous type rather than the other pathological types. (p<0.05, significant on the chi-square test)

Supplemental figure 6


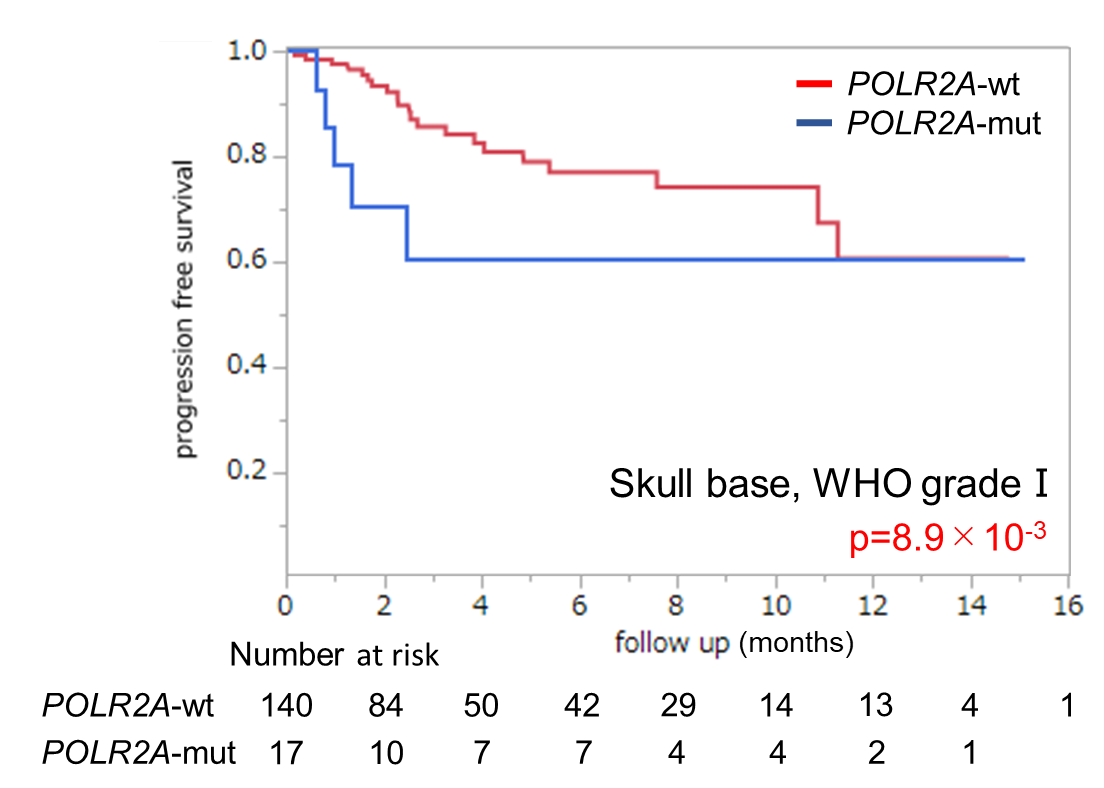


Supplemental Figure 6 Kaplan-Meier plots of time to recurrence based on the *POLR2A* mutation

Comparison of skull-base and WHO grade I tumors in patients harboring wild type and mutant *POLR2A*

Progression-free survival of patients harboring *POLR2A* mutations was worse than those of patients harboring wild type *POLR2A* (p<0.05, significant on the log-rank test)

Supplemental Figure 7


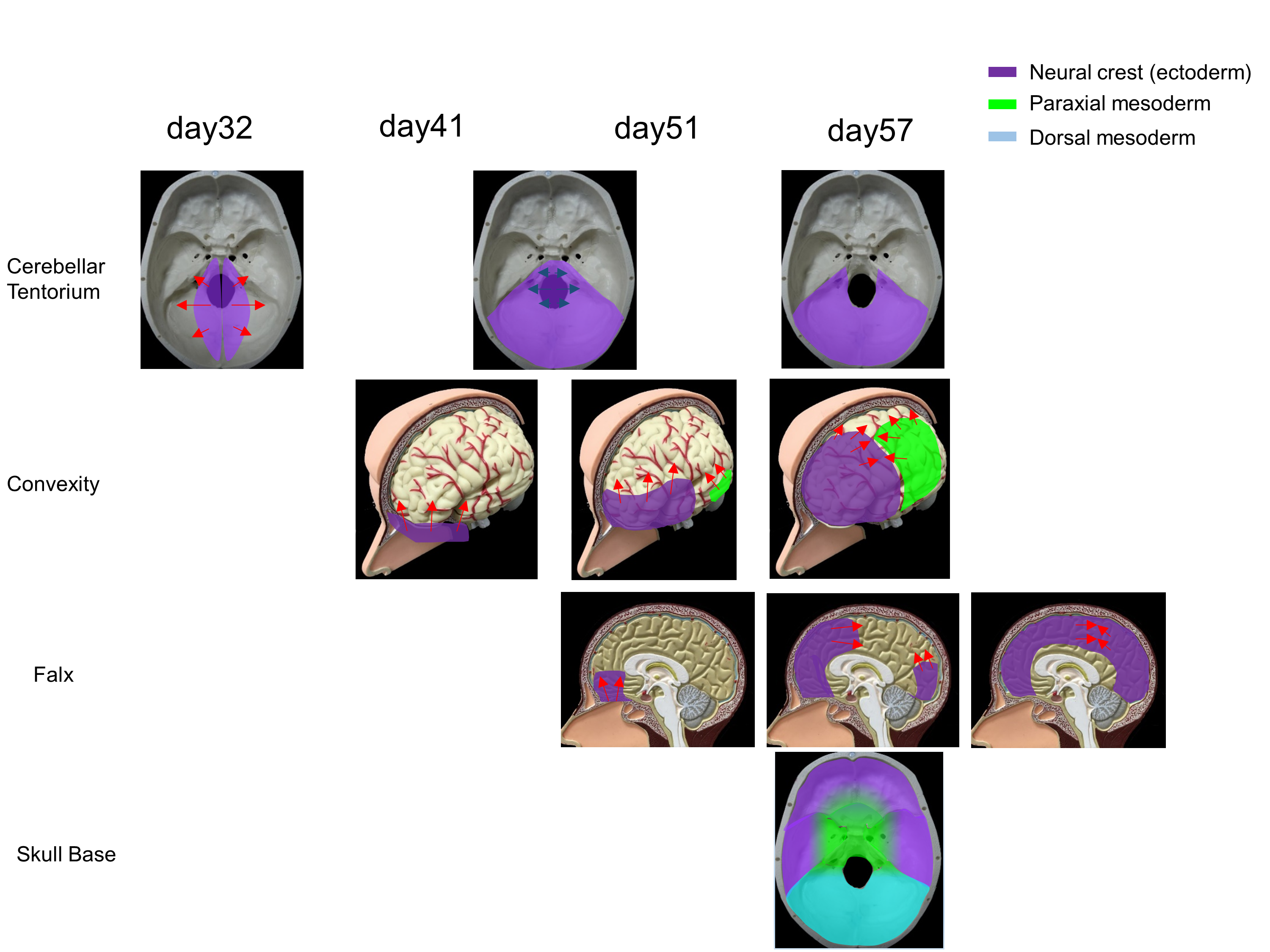


Supplemental Figure 7 Normal meningeal and dural development

Normal development of the tentorium cerebelli, convexity meninges, falx, and skull-base dura are depicted in each fetal period. Purple indicates the meninges derived from the neural crest; green, paraxial mesoderm; blue, dorsal mesoderm.

Figure generated using Microsoft PowerPoint 2016, [https://www.microsoft.com](https://www.microsoft.com/).

Supplemental table 1 Risk factors for tumor recurrence in skull-base and WHO grade I tumors

|  |  | Univariate |  |  | Multivariate |  |
| --- | --- | --- | --- | --- | --- | --- |
|  | Hazard ratio | *p* value | 95% CI | Hazard ratio | *p* value | 95% CI |
| Female | 1.00 | 1.00 | 0.42-2.38 |  |  |  |
| Age≥65 | 0.69 | 0.55 | 0.20-2.34 |  |  |  |
| Simpson grade 1-3 | 0.18 | 3.0×10^-5^ | 0.08-0.41 | 0.25 | 1.5×10^-3^ | 0.11-0.59 |
| MIB-1 LI**≥**3 | 2.99 | 0.01 | 1.27-7.05 | 3.26 | 0.01 | 1.27-8.40 |
| Neural crest | 1.12 | 0.69 | 0.64-1.96 |  |  |  |
| Paraxial mesoderm | 1.20 | 0.54 | 0.67-2.14 |  |  |  |
| Dorsal mesoderm | 0.43 | 0.26 | 0.23-1.39 |  |  |  |
| *AKT1* mutation | 0.67 | 0.51 | 0.20-2.23 |  |  |  |
| *KLF4* mutation | 0.77 | 0.73 | 0.18-3.28 |  |  |  |
| *POLR2A* mutation | 2.44 | 0.07 | 0.92-6.50 | 2.80 | 0.04 | 1.16-9.53 |
| *NF2* mutation or 22q loss | 1.21 | 0.62 | 0.56-2.63 |  |  |  |

LI indicates labelling index and CI, confidence interval
